# Determinants of Periodic Health Examination Uptake: Insights From a Jordanian Cross-Sectional Study

**DOI:** 10.2196/57597

**Published:** 2025-02-05

**Authors:** Abdul Aziz Tayoun

**Affiliations:** 1 School of Medicine, Department of Family and Community Medicine, Jordan University, Qween Rania Street, Amman, 11942, Jordan, 962 778435800

**Keywords:** periodic health examination, PHE, preventive health services, routine health checkups, Jordan, cross-sectional study

## Abstract

**Background:**

Routine periodic health examinations (PHEs) for adults who are asymptomatic are included in clinical preventive services. They aim to prevent morbidity and mortality by identifying modifiable risk factors and early signs of treatable diseases. PHEs are a standard procedure in primary health care worldwide, including in Jordan. The country is undergoing an epidemiological transition toward noncommunicable diseases, which are the leading causes of morbidity and mortality. The prevalence of smoking is among the highest in the world, with escalating rates of obesity and physical inactivity. Notably, hypertension and diabetes are the most prevalent diseases.

**Objective:**

This study aims to determine the extent to which individuals in Jordan participate in PHEs and to evaluate the various factors related to sociodemographics, health, knowledge, and behavior that influence this participation.

**Methods:**

This study used a cross-sectional design and includes 362 participants 18 years or older residing in Jordan. A convenience sampling method was used, and data were collected through a hybrid web-based and face-to-face questionnaire. The analysis involved the application of logistic regression through SPSS to investigate the relationship between various influencing factors and the uptake of PHEs.

**Results:**

Our study indicated that only 98 of the 362 (27.1%, 95% CI 22.8%-31.9%) participants underwent PHEs within the last 2 years. Noteworthy predictors of PHE uptake among Jordanians included recent visits to a primary health care facility within the previous year (adjusted odds ratio [AOR] 4.32, 95% CI 2.40‐7.76; *P*<.001), monthly income (*P*=.02; individuals with a monthly income of 1500‐2000 JD displayed more than five times the odds of undertaking PHEs than those with a monthly income <500 JD; AOR 5.74, 95% CI 1.32‐24.90; *P*=.02; those with a monthly income of more than 2000 JD exhibited even higher odds; AOR 9.81, 95% CI 1.73‐55.55; *P*=.02; a currency exchange rate of 1 JD=US $1.43 is applicable), and knowledge levels regarding PHEs and preventive health measures (AOR 1.23, 95% CI 1.03‐1.47; *P*=.007). These variables emerged as the strongest predictors in our analysis, shedding light on key factors influencing PHE uptake in the population. Contrary to other research, our study did not find any statistically significant association between gender (*P*=.33), smoking status (*P*=.76), marital status (*P*=.52), health status self-evaluation (*P*=.18), seasonal influenza vaccination (*P*=.07), combined health behavior factors (*P*=.34), and BMI (*P*=.76) and PHE uptake.

**Conclusions:**

PHE uptake is notably low in Jordan. Critical determinants of this uptake include recent visits to a primary health care facility within the previous year, monthly income, and knowledge levels regarding PHEs and preventive health services. To enhance PHE uptake, there is a critical need to integrate PHEs with primary health care services, increase awareness about PHEs, and offer free preventive services, particularly for those at high risk.

## Introduction

### Background

Routine periodic health examinations (PHEs) for adults who are asymptomatic are integral to primary health care practice. These examinations involve clinical preventive services administered by primary health care clinicians to individuals without signs or symptoms of illness, constituting a routine health care process. The goal of these examinations is to prevent morbidity and mortality proactively, this is achieved by identifying modifiable risk factors and detecting early signs of treatable diseases [1].

The health belief model (HBM) was conceptualized to elucidate why individuals are reluctant to engage in disease prevention programs and health checkups. As a crucial predictive framework, the HBM aids in understanding various health-related behaviors, including smoking, exercise, patient roles, and use of medical services [2].

Integrating with the HBM, health beliefs are defined as personal convictions associated with perceiving and managing specific diseases. These beliefs encompass key elements: perceived sensitivity, perceived severity, perceived benefit, perceived barrier, and cue to action [3].

### Literature Review

A systematic review recently published in the *Canadian Family Physician Journal* aimed to assess the reasons for visits to primary health care clinics. Clinicians participating in the review identified routine health maintenance as the third most prevalent reason for individuals seeking consultations with primary health care physicians. This ranking positioned routine health maintenance after upper respiratory tract infections and hypertension, highlighting the significant role of primary health care practitioners in motivating individuals to engage with preventive health services [4].

A study conducted among undergraduate students in a Nigerian health science college found that 91.2% of participants demonstrated awareness of PHEs. However, the actual participation in PHEs was notably low at 28.4%. The primary obstacles to uptake were identified as insufficient time, religious considerations, duration of education, perceived susceptibility to diseases, financial constraints, apprehension about the results, and a general lack of interest [5].

A nationwide study in Saudi Arabia revealed that 22.9% of participants 15 years or older had undergone a PHE in the preceding 2 years. The probability of receiving a PHE during this period exhibited positive correlations with various factors—including age; educational attainment; marital status; regular consumption of five servings of fruits and vegetables daily; and diagnoses such as prediabetes, diabetes, or hypercholesterolemia—visit to a health care setting within the last 2 years due to illness or injury [6].

### Rationale and Significance of the Study

Jordan, classified as an upper middle–income country, spans an area of 89,318 square kilometers and is divided into four provinces and 12 governorates. The population has grown substantially, increasing from 5.4 million in 2003 to over 11.5 million in 2023. This demographic shift can be attributed mainly to the influx of refugees and a relatively high birth rate [7,8].

The country is undergoing a notable epidemiological transition characterized by a rising prevalence of noncommunicable diseases (NCDs). These diseases are responsible for approximately 78% of deaths, establishing themselves as the primary cause of mortality and morbidity among the Jordanian population. Key risk factors contributing to the burden of NCDs include tobacco use, with a prevalence of about 50% (including e-cigarettes and shisha). One-quarter of the population reports insufficient physical activity and approximately 60% are classified as overweight or obese. Additionally, 22% of the population has hypertension, 14% has diabetes, and about 18% has depression [9].

### Goals of This Study

This profile underscores a pressing concern regarding the country’s high risk of NCDs. There is a need for evidence-based preventive health measures to curb the progression of NCDs and their associated risk factors. If conducted according to evidence-based guidelines, PHEs can effectively control communicable diseases and NCDs. Recognizing the urgency of the situation, gathering data on the uptake rate of PHEs, and identifying the factors influencing this uptake is imperative. The absence of previous studies on the uptake of PHEs in Jordan underscores the necessity for comprehensive research. Our study aims to estimate the uptake of PHEs among Jordanians while concurrently investigating various sociodemographic, health status, knowledge, and behavioral factors that play a role in influencing this uptake. The findings from this research will not only contribute valuable insights into the current scenario but also guide educational and promotional activities to encourage citizens to use preventive health services. In doing so, we strive to fill a crucial gap in existing knowledge and provide a foundation for evidence-based strategies to enhance public health in the country.

## Methods

### Recruitment

This descriptive cross-sectional study was conducted using an anonymous web-based Google Forms questionnaire between March 15 and May 1, 2023. Due to the lack of resources, a convenience sampling method was used to recruit participants. Jordanian residents aged ≥18 years who agreed to participate in our study were considered eligible. The research uses a questionnaire with five key domains: sociodemographic, health status, PHE uptake history, knowledge about PHEs, and health behaviors based on the HBM. This questionnaire was sent through the WhatsApp and Facebook platforms to participants, who were encouraged to share them with their family members. In addition, collecting data through face-to-face interviews targeted clients of grand malls, mosques, and pharmacies, supplementing the web-based data collection.

The study adopted a stratified proportional sampling strategy across four provinces of Jordan. This approach is carefully extended to maintain a balance in gender and nationality among participants. The initial page of the web-based questionnaire explicitly outlines the study’s objectives and provides detailed instructions on how to complete the questionnaire. This effort was complemented by the researcher’s availability to answer questions, ensuring participants’ queries or doubts were promptly addressed.

### Sampling Method

The following inclusion and exclusion criteria were used:

- Inclusion criteria: any citizen regardless of nationality, 18 years or older, and residing in Jordan
- Exclusion criteria: persons younger than 18 years and individuals who declined to participate in the study

We recruited 362 respondents, aiming to provide a representative sample that reflects the entire population of Jordan in terms of district, age, sex, and nationality. The convenience sample size of 362 was calculated using the sample size formula for proportions:


$$N=Z_{\propto /2}^{2}\frac{P\left(1-P\right)}{D^{2}}$$

This calculation considered a study conducted in Saudi Arabia, where approximately 34% of the population underwent PHEs [10]. The chosen values for statistical significance (α error) and margin of error (D) were .05% and 5%, respectively. As a result, the calculated sample size required for the survey was 345 respondents.

### Questionnaire Development

The PHE questionnaire (Multimedia Appendix 1), comprising 36 questions across five domains, was developed following an extensive literature review [10-14]. The questionnaire’s five domains are as follows:

1. Sociodemographic (9 items): inquires about relevant sociodemographic variables of participants
2. Health status and risk factors (7 items): explores participants’ health status and associated risk factors
3. PHE uptake (4 items): focuses on the outcome variable of PHE uptake
4. Knowledge about PHE and preventive health services (8 items): assesses knowledge using a 3-option scale (agree, don’t agree, I don’t know). The items are scored, with correct answers receiving a score of 1 and incorrect or I don’t know responses scoring 0. The total score ranges from 0 to 8, with higher scores indicating more significant knowledge of health checkups and preventive measures. The Cronbach α, estimated during the pilot phase with 25 participants, was 0.68.
5. Health behaviors toward PHE based on the HBM (6 items): measures health behaviors using a 5-point Likert scale ranging from 1 (strongly disagree) to 5 (strongly agree). The total score ranges from 6 to 30, with higher scores indicating more positive health beliefs for each item. The Cronbach α for health behaviors toward PHEs during the pilot testing phase was 0.74, demonstrating acceptable internal consistency.

The questionnaire was translated into Arabic for comprehensibility and then back to English with the assistance of an expert translator. This rigorous process ensures the questionnaire’s clarity and accuracy across languages.

### Statistical Analysis

The primary outcome variable is the uptake of PHEs in Jordan, categorized as a dichotomous (yes or no) variable. The independent variables encompass sociodemographics, health status, knowledge, and health behavioral factors. Records with missing data were excluded to ensure the integrity of the analysis. Data was analyzed using SPSS, version 26.0 (IBM Corp).

Participant characteristics were examined using counts, percentages, means, and SDs through descriptive statistics. Graphs and tables were used as needed for visual representation. A 95% CI was calculated using appropriate methods, and a 2-sided *P *value <.05 was considered statistically significant.

A binary logistic regression test was used to study the association between the binary outcome variable and the various continuous and nominal predictor variables. Multivariate logistic regression analysis was used to examine the relationship between the uptake of PHEs and various independent covariables to adjust for confounding.

A hierarchical block-wise logistic regression model was also constructed to identify the most potent predictor variables. This comprehensive approach blends descriptive, inferential, and multivariate statistical techniques to provide a thorough understanding of the factors influencing the uptake of PHEs in Jordan.

### Ethical Considerations

Before the formal survey, the study protocol was approved by the Jordan University Ethics Committee (approval 13‐2023) and the Jordan University Hospital Ethics Committee (approval 10/2023/4560). The questionnaire was designed to be anonymous and voluntary, and respondents were informed that submission of the questionnaire implied informed consent. The data were kept confidential, and the results did not identify the respondents personally. Contact information for the researcher was provided for clarification purposes. No compensation was provided to participants.

## Results

A total of 365 individuals participated in the study between March and April 2023, with a response rate of 99%; 3 participants were excluded (one was younger than 18 years, and the other two did not complete the questionnaire), leaving 362 participants for analysis.

### Descriptive Statistics

The demographic characteristics of participants are summarized in Table 1. The mean age was 38.2 (SD 14.6, range 18-88) years. Of the 362 participants, there were slightly more male (n=185, 51.1%) than female participants. Approximately 230 (63.5%) were married, 270 (74.6%) were Jordanians, and 202 (55.8%) held a university degree. Most participants (n=225, 62.1%) reported a monthly income of less than 500 JD (a currency exchange rate of 1 JD=US $1.43 is applicable), with half lacking health insurance.

Regarding health status, Table 2 shows that 240 (66.3%) participants reported good or excellent health, 78 (21.5%) had a chronic disease, and 200 (55.2%) visited a primary health care clinic in the past year. Additionally, 191 (52.8%) participants were current smokers.

Regarding PHEs, only 98 of the 362 (27.1%, 95% CI 22.8%‐31.9%) participants underwent a medical checkup in the last 2 years.

**Table 1.** Sociodemographic characteristics of participants (N=362).

Characteristic		Participants, n (%)
Gender		
Male		185 (51.1)
Age group (years)		
18‐29		122 (33.7)
30‐39		90 (24.9)
40‐49		70 (19.3)
50‐59		41 (11.3)
≥60		39 (10.8)
Marital status		
Married		230 (63.5)
Single		101 (27.9)
Divorced		14 (3.9)
Widowed		17 (4.7)
Monthly income (JD)^a^		
<500		225 (62.1)
500‐999		93 (25.7)
1000‐1499		26 (7.2)
1500‐1999		10 (2.8)
≥2000		8 (2.2)
Educational level		
Elementary school		42 (11.6)
Secondary school		118 (32.6)
University		166 (45.9)
Postgraduate		36 (9.9)
Province of residence		
Amman		151 (41.7)
Central Jordan		82 (22.7)
North Jordan		100 (27.6)
South Jordan		29 (8.0)
Nationality		
Jordanians		270 (74.6)
Syrians		47 (13.0)
Palestinians		22 (6.1)
Egyptians		18 (5.0)
Iraqis		5 (1.4)

a A currency exchange rate of 1 JD=US $1.43 is applicable.

**Table 2.** Health characteristics of participants in the study.

Variable		Participants, n (%)
Visiting a primary health care facility within the previous year		
Yes		200 (55.2)
No		162 (44.8)
Noncommunicable diseases		
Yes		78 (21.5)
No		284 (78.5)
Smoking		
Smoker		191 (52.8)
Not smoker		171 (47.2)
Health insurance		
Insured		183 (50.6)
Not insured		179 (49.4)
Seasonal flu vaccination		
Yes		60 (16.6)
No		302 (83.4)
Health status self-evaluation		
Poor		9 (2.5)
Fair		25 (6.9)
Good		88 (24.3)
Very good		136 (37.6)
Excellent		104 (28.7)
BMI≥25		
Yes		223 (61.6)
No		139 (38.4)
Physical activity		
Yes		108 (29.8)
No		254 (70.2)

### Logistic Regression Analysis

**Figure 1.**
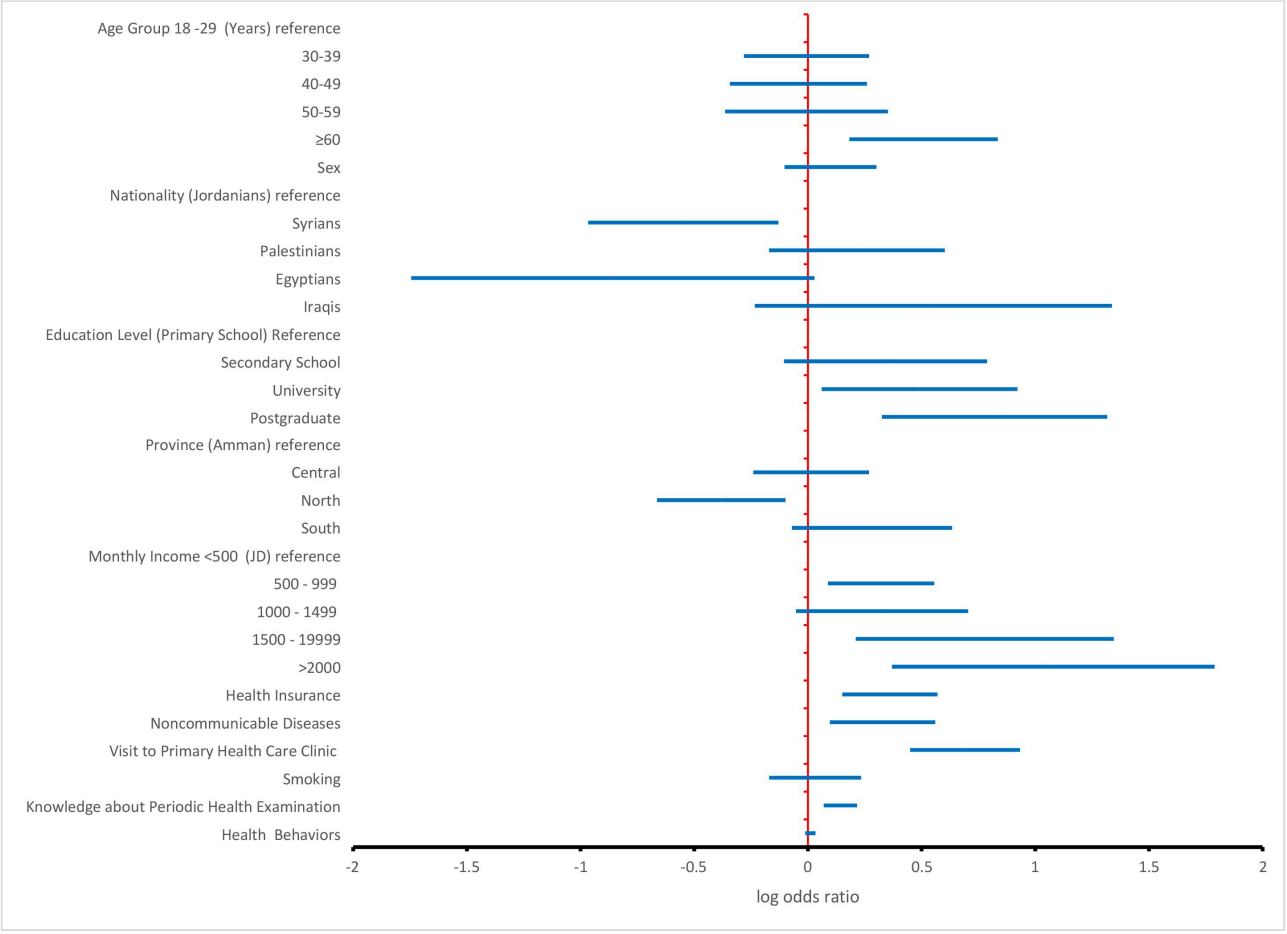
Univariate logistic regression analysis for predictor factors of periodic health examination uptake, Jordan 2023. A currency exchange rate of 1 JD=US $1.43 is applicable.

The forest plot in Figure 1 highlights several significant findings from the analysis of the predicting factors’ association with PHE uptake.

Age was found to be a significant determinant of PHE uptake: with each additional year of age there, is a 2.2% increase in the odds of undertaking PHEs (odds ratio [OR] 1.022, 95% CI 1.006‐1.038; *P=*.006). Nationality also proved to be a factor, with Syrians demonstrating a lower frequency of PHE uptake. The odds of Syrians undergoing PHEs were 0.283 compared to Jordanians (OR 0.28, 95% CI 0.11‐0.74; *P*=.01). Education level exhibited a strong association, with postgraduates displaying more than 6 times the odds of undertaking PHE than individuals with only primary school education (OR 6.62, 95% CI 2.12‐20.71; *P*=.001). Health care workers displayed more than 12 times the odds of undergoing PHEs than general employees (OR 12.28, 95% CI 4.69‐32.19; *P*<.001). Individuals earning more than 2000 JD monthly had 12 times greater odds of receiving PHEs compared to those with a monthly income of less than 500 JD (OR 12.00, 95% CI 2.34‐61.45; *P*=.003). Health insurance emerged as a significant facilitator of PHE uptake. Insured participants demonstrated more than 2 times the odds of undertaking PHEs than noninsured individuals (OR 2.30, 95% CI 1.42‐3.71; *P*=.001). People with chronic diseases have more than twice the odds of undertaking PHEs than those without chronic diseases (OR 2.3, 95% CI 1.258‐3.629; *P*=.005). Visits to a primary health care clinic in the past year significantly impacted PHE uptake. Those who had visited had 5 times the odds of PHE uptake compared to those who did not visit a primary health care facility in the past year (OR 4.91, 95% CI 2.82‐8.57; *P*<.001). Participants who were physically active had 1.65 times the odds of undertaking PHEs than those without enough physical activity (OR 1.65, 95% CI 1.01-2.69; *P*=.046). Finally, for every extra point in knowledge about PHEs, there is a 39% increase in PHE uptake (OR 1.39, 95% CI 1.18‐1.64; *P*<.001).

On the other hand, several variables were not associated with PHE uptake. These included gender (*P*=.33), smoking status (*P*=.76), marital status (*P*=.52), health status self-evaluation (*P*=.18), seasonal influenza vaccination (*P*=.07), combined health behavior factors (*P*=.34), and BMI (*P*=.76).

### Adjusted Logistic Regression Model

After meticulously adjusting for confounding variables and carefully selecting clinically and statistically significant factors, we successfully constructed a logistic regression model using the hierarchical block-wise method. This refined model, depicted in Table 3, encapsulates three variables that significantly influence the uptake of PHEs.

**Table 3.** Logistic regression model for most significant predictor factors for periodic health examination uptake, Jordan 2023.

Variable	*P* value	Adjusted odds ratio (95% CI)
Visiting a primary health care facility	<.001	4.315 (2.40-7.76)
Knowledge about periodic health examinations	.02	1.230 (1.03-1.47)
Monthly income (JD)^a^	.07	
<500 (reference)	—^b^	1.00
500‐999	.07	1.71 (0.96-3.02)
1000‐1499	.11	2.18 (0.84-5.66)
1500‐1999	.02	5.74 (1.32-24.90)
≥2000	.01	9.81 (1.73-55.55)

a A currency exchange rate of 1 JD=US $1.43 is applicable.

b Not applicable.

#### Visit to Primary Health Care Facilities in the Past Year

Visiting primary health care facilities within the past year exhibited a substantial impact on PHE uptake. These individuals demonstrated more than 4 times the odds of undertaking PHEs compared to those who did not visit a primary health care facility within the same time frame (adjusted OR [AOR] 4.32, 95% CI 2.40‐7.76; *P*<.001).

#### Income Level

Individuals with a monthly income of 1500‐2000 JD displayed more than five times the odds of undertaking PHEs than those with a monthly income of less than 500 JD (AOR 5.74, 95% CI 1.32‐24.90; *P*=.02). Furthermore, those with a monthly income of more than 2000 JD exhibited even higher odds (AOR 9.81, 95% CI 1.73‐55.55; *P*=.02).

#### Health Knowledge

The analysis indicates that for every point increase in PHE knowledge, the likelihood of individuals opting for PHEs increases by 23% (AOR 1.23, 95% CI 1.03-1.47; *P*=.02).

## Discussion

### Principal Findings and Comparison With Other Studies

Of the 362 participants, only 98 (27.1%, 95% CI 22.8%-31.9%) had undergone a PHE in the past 2 years. Some significant predictors included recent visits to a primary health care facility the previous year, monthly income, knowledge about PHEs, and preventive health measures. Other nonsignificant factors were gender, marital status, smoking status, and BMI, which did not emerge as being significantly associated with the uptake of PHEs.

Interestingly, the uptake rate observed in our study is comparable to that reported in studies conducted in Saudi Arabia [6,10] and Nigeria [12]. In contrast, this rate notably fell below those reported in studies conducted in the United States [1], the United Kingdom [13], and Switzerland [15].

The most influential determinant for the uptake of PHEs found in our study was a visit to a primary health care facility in the past year. Our findings again were consistent with those from several other studies [6,16,17]. Notably, those who had visited any primary health care clinic in the previous year were found to be five times more likely to undertake PHEs compared to those who had not visited such clinics in the same time frame. This association was statistically significant even after adjusting for other relevant factors, thus underlining its strength. The second most important factor influencing the uptake of PHEs was monthly income. This finding agrees with results from other sources [1,12,14,17-21]. The influence of monthly income on the uptake of PHEs reflects how socioeconomic issues can affect health care–seeking behavior. There is a great need for focused efforts or an intervention policy that addresses these issues. Knowledge about PHEs was the third most influential factor. The findings are in agreement with those of previous studies [22-24] and underline the role of informed choice in health care use. This paper should, however, state that knowledge of PHEs was associated with other factors such as educational level and occupation. However, adjustment for these factors associated with knowledge of PHEs did not reduce the strength of the association with knowledge and PHE uptake.

More variables were positively associated with the uptake of PHEs. The older the age, the better the PHE uptake, which agrees with other studies’ findings [13,17,19]. This may indicate that with increased age, people are likely to undergo regular health checkups, either because of the higher burden of NCDs in older age or maybe because more attention is paid to preventive measures with increased age. Individuals of Syrian nationality were found to be less likely to undergo PHEs than Jordanians. Economic factors may explain this difference, emphasizing the need for targeted interventions to ensure equitable access to preventive health care services among diverse populations. There was a strong association between education and PHE uptake, evidenced by a substantial increase in PHE uptake corresponding to higher levels of education. This finding is similar to results from other studies [17,21,25]. Compared to employees in general, health workers and retirees were more likely to undergo PHEs. This may be because health care workers are more aware of the importance of preventive health. Age can serve as a confounder for retired people because it may affect retired status and PHE uptake.

The health-related factors identified to be associated with PHE uptake in our study, and supported by other studies, include the presence of chronic diseases [6,14,18,22,26], being insured [17,21,25,27,28], and engagement in physical activity [1].

Other factors showed no significant association with the uptake of PHEs. For example, one nonsignificant factor was sex, which contrasts many studies indicating that females are more willing to participate in PHEs than males [6,13,15,20]. Being married has often been linked to higher PHE uptake in previous studies but not in our study [1,13-15,19,29,30]. Surprisingly, smoking status was not associated with the uptake of PHEs; several studies in the past have argued that smokers are less likely than nonsmokers to undergo PHEs [11,13,15,20,29]. Our study did not find any clear association between combined behavioral factors and the uptake of PHEs, although many studies identify such associations [3,11,14,20,30,31]. This is possibly because of the suitability of the questionnaires to the Jordanian population or problems with participants understanding.

### Strengths of the Study

This study is the first of its kind to investigate the uptake of PHEs in Jordan and hence addresses an important gap in existing knowledge. Given that this is the first study on this topic, it has contributed quite substantially to the understanding of PHE uptake in the country. The statistical analysis approach adopted in this study is broad and solid, using descriptive, inference, and multivariate statistical techniques. This approach leads to a deeper analysis and more reliable findings. The study also managed to identify the significant predictors of PHE uptake.

### Limitations of the Study

One of the primary limitations is its cross-sectional design, which restricts the ability to establish causality between the different predictor factors and PHE uptake. To address this issue, future research could adopt a longitudinal approach, providing a better understanding of how these predictors influence PHE uptake. Another limitation relates to the sampling method. The study used a convenience sampling strategy, which may have introduced selection bias, and the web-based survey format could lead to measurement bias. To decrease the chances of bias, we used a stratified sampling method, taking into account population size and stratifying participants by gender, age group, and nationality across the four provinces of Jordan. Additionally, a hybrid approach integrating both web-based and face-to-face interviews, and collecting data from various settings such as social media platforms, grand malls, mosques, and pharmacies helped ensure a more representative sample. The author’s availability for clarifications via WhatsApp and email also aimed to reduce potential measurement biases during data collection. The third limitation concerns the survey instrument itself. The comprehensiveness and relevance of the questionnaire to the Jordanian population might not have been fully ensured. To address this issue, a pilot study with 25 participants was conducted, and the questionnaire was revised based on their feedback and reliability measures. Lastly, the study’s results may have limited generalizability beyond the population of Jordan. To enhance the applicability of the findings to broader populations, future research should consider a more diverse sample by including other countries. This would provide a more comprehensive understanding of PHE uptake within and outside Jordan.

### Future Directions

First, we established that recent visits to primary health care facilities were the strongest predictor of PHE uptake. From this, we recommend incorporating preventive health services into existing primary health care services to enhance accessibility and efficiency. This may take the form of incentivizing both health caregivers and patients. Second, economic issues can be resolved by suggesting the provision of all preventive services free of cost at primary health care centers. Private health insurance companies can also facilitate this endeavor by covering preventive services like PHEs within the realm of their service provision so that people can have better access to these services. More importantly, public awareness will have to increase. The positive correlation between knowledge of PHEs and their uptake points to a need for more organized and evidence-based awareness campaigns. Another issue involves the study’s findings on behavioral factors. The study did not find a significant relationship between behavioral factors and PHE uptake, contradicting findings from other contexts. To better understand these results, future research could involve a more detailed investigation into the cultural and societal influences on health behaviors in Jordan, which may help clarify why these factors did not show the expected association. It is also recommended that further studies, especially on smoking as a predictor factor for PHE uptake, be done in detail to understand how to best address these areas in future studies.

### Conclusion

Our study has highlighted the low level of PHE uptake in Jordan. This paper identified visitation to primary health care facilities in the past year, monthly income, and knowledge about PHEs and preventive health services as the major predictors influencing the likelihood of undergoing PHEs. The association of regular visits to primary health care facilities with higher uptake of PHEs suggests that PHEs should be integrated with the available services at primary health care facilities. These findings also suggest that targeted interventions should be implemented to enhance awareness and knowledge of the value of preventive health practices among the Jordanian population, particularly for patients with lower income status.

## Supplementary material

10.2196/57597 Multimedia Appendix 1 Questionnaire in English.

10.2196/57597 Multimedia Appendix 2 ChatGPT’s draft.

10.2196/57597 Checklist 1 STROBE (Strengthening the Reporting of Observational Studies in Epidemiology) checklist.

## Abbreviations

AOR: adjusted odds ratio
HBM: health belief model
NCD: noncommunicable disease
OR: odds ratio
PHE: periodic health examination
