# Supplementary material for: Determinants of Periodic Health Examination Uptake: Insights From a Jordanian Cross-Sectional Study
Source: JMIRx Med. 2025 Feb 5;6:e57597. doi: 10.2196/57597 (PMC11822400; doi:10.2196/57597)
Supplement: Multimedia Appendix 1 [file xmed-v6-e57597-s001.docx]

Questionnaire to Measure the Desire of Jordanians for Regular Health Check-ups

Dr. Abdul Aziz Tayon / Consultant in Community Medicine and Public Health

A. Consent to Participate

- This regular health check-up is a medical examination performed on individuals who do not exhibit symptoms at the time of the examination. Its purpose is to detect diseases at an early stage and identify risk factors associated with communicable and non-communicable diseases for treatment.

This study aims to measure the proportion of Jordanians who have undergone regular health check-ups in the past two years and to identify the factors that encourage or hinder participation.

- This questionnaire will take approximately 4 minutes to complete. Participation is voluntary, and you can withdraw anytime by closing the browser.

- For inquiries, contact the researcher via email at aatayoun@yahoo.com.

1. I acknowledge that I have read the above information and agree to participate in the survey. I also confirm that I am over 18 years old:

- I agree to participate

- I do not agree to participate

B. Personal Information

2. Age in years : ……………………

3. Gender:

- Male

- Female

4. *Marital Status:

- Married

- Single

- Widowed

- Divorced

5. Nationality:

- Jordanian

- Palestinian

- Syrian

- Iraqi

- Egyptian

- Other

6. Educational Level:

- Primary school

- Secondary school

- Bachelor's

- Postgraduate

7. Residence:

- Amman

- Central Jordan

- Northern Jordan

- Southern Jordan

8. Employment Status:

- Employed

- Retired

- Student

- Healthcare worker

- Unemployed

- Other

9. Monthly Income:

- Less than 500 Dinars

- 500-1000 Dinars

- 1000-1500 Dinars

- 1500-2000 Dinars

- More than 2000 Dinars

10. Do you have health insurance?

- Yes

- No

C. Health Status

11. How would you rate your health?

- Excellent

- Very Good

- Good

- Fair

- Poor

12. Do you have any chronic diseases (e.g., hypertension, diabetes, asthma, etc.)?

- Yes

- No

- Don't know

13. Have you visited any medical clinic or hospital in the past year?

- Yes

- No

14. Do you smoke (including hookah and electronic cigarettes)?

- Yes, daily

- Yes, occasionally

- Former smoker

- No, never smoked

15. Weight (kg) : ……………

16. *Height (cm):………………

17. Do you engage in any physical activity, including exercise, for at least half an hour daily?

- Yes

- No

18. Have you received the flu vaccine this year?

- Yes

- No

D. Regular Health Check-up

19. In the past two years, have you visited a doctor for a regular health check-up (excluding visits for medical symptoms)?

- Yes

- No

20. Which specialty did you visit for your regular health check-up?

- General Medicine

- Family Medicine

- Internal Medicine

- Never had a regular health check-up

21. If you have never had a regular health check-up, what is the reason?

- Not convinced of its importance

- Financial cost

- Lack of time

- Health insurance does not cover it

- Fear of discovering a serious illness

- Lack of trust in medical staff

- I have had a check-up before

- Other

22. *Do you intend to have a regular health check-up in the future?

- Yes

- No

- Undecided

E. Knowledge about Regular Health Check-ups

- Answer the following questions that measure your knowledge about regular health check-ups (Agree, Disagree, or Don't know)

23. Regular health check-ups are conducted on people without symptoms:

- Agree

- Disagree

- Don't know

24. Sitting with someone who smokes (cigarettes, hookah, or e-cigarettes) does not affect my health as long as I do not smoke:

- Agree

- Disagree

- Don't know

25. Heart diseases are the leading cause of death in Jordan:

- Agree

- Disagree

- Don't know

26. Treating type 2 diabetes begins with a healthy lifestyle (balanced diet and exercise):

- Agree

- Disagree

- Don't know

27. Blood pressure should be measured annually even if there are no symptoms:

- Agree

- Disagree

- Don't know

28. Exercising once a week is enough to prevent diseases:

- Agree

- Disagree

- Don't know

29. There is a vaccine to prevent cervical cancer:

- Agree

- Disagree

- Don't know

30. Hepatitis B is transmitted through contaminated food:

- Agree

- Disagree

- Don't know

F. Health Behaviors

- Answer the following questions on a scale from 1-5 (1: Strongly Disagree to 5: Strongly Agree)

31. Even if my health is good and I have no symptoms, I should see a doctor annually or every few years to check on my health:

1 Strongly Disagree

2 Disagree

3 Neutral

4 Agree

5 Strongly Agree

32. I am not afraid of discovering a disease through regular health check-ups because early detection is better than late discovery:

1 Strongly Disagree

2 Disagree

3 Neutral

4 Agree

5 Strongly Agree

33. Regular health check-ups make me feel reassured about my health:

1 Strongly Disagree

2 Disagree

3 Neutral

4 Agree

5 Strongly Agree

34. Many diseases, if detected early, are curable.

1 Strongly Disagree

2 Disagree

3 Neutral

4 Agree

5 Strongly Agree

35. Many diseases, if not treated, lead to serious complications or death.

1 Strongly Disagree

2 Disagree

3 Neutral

4 Agree

5 Strongly Agree

36. I can allocate enough time to perform routine health screening.

1 Strongly Disagree

2 Disagree

3 Neutral

4 Agree

5 Strongly Agree
