# Supplementary material for: Determinants of Periodic Health Examination Uptake: Insights From a Jordanian Cross-Sectional Study
Source: JMIRx Med. 2025 Feb 5;6:e57597. doi: 10.2196/57597 (PMC11822400; doi:10.2196/57597)
Supplement: Multimedia Appendix 2 [file xmed-v6-e57597-s002.docx]

**Determinants of Periodic Health Examination Uptake: Insights from a Cross-Sectional Study in Jordan**

**Abstract**

**Background**
Routine periodic health examinations (PHEs) for asymptomatic adults involve clinical preventive services aimed at preventing morbidity and mortality by identifying modifiable risk factors and early signs of treatable diseases. PHEs are a standard procedure in primary healthcare worldwide, including in Jordan, a country undergoing an epidemiological transition toward non-communicable diseases (NCDs). These diseases are now the leading causes of morbidity and mortality. Jordan also has one of the highest smoking rates globally, alongside escalating rates of obesity and physical inactivity. Notably, hypertension and diabetes mellitus are the most prevalent conditions.

**Objectives**
This study aims to determine the extent of participation in periodic health examinations among individuals in Jordan and to evaluate the sociodemographic, health-related, knowledge, and behavioral factors that influence this participation.

**Methods**
A cross-sectional study was conducted with 362 participants aged 18 years or older residing in Jordan, achieving a 99% response rate. A convenient sampling method was used, and data were collected through a hybrid online and face-to-face questionnaire. Logistic regression analysis was performed using SPSS to explore the relationship between various influencing factors and the uptake of periodic health examinations (PHEs).

**Results**
The study found that only 98 out of 362 participants (27.1%) had undergone a PHE within the last two years, with a 95% confidence interval of 22.8% to 31.9%. Significant predictors of PHE uptake included recent visits to a primary healthcare facility within the last year (P < .001, AOR = 4.32, 95% CI 2.40–7.76) and monthly income (P = .02). Individuals earning 1,500–2,000 JD had more than five times the odds of undertaking a PHE compared to those earning less than 500 JD (P = .02, AOR = 5.74, 95% CI 1.32–24.90), while those with a monthly income of more than 2,000 JD exhibited even higher odds (P = .02, AOR = 9.81, 95% CI 1.73–55.55). Additionally, knowledge levels regarding PHEs and preventive health measures (P = .007, AOR = 1.23, 95% CI 1.03–1.47) were significant predictors.

Contrary to findings from other studies, no statistically significant associations were found between gender (P = .33), smoking status (P = .76), marital status (P = .52), self-evaluated health status (P = .18), seasonal influenza vaccination (P = .07), combined health behavior factors (P = .34), or BMI (P = .76) and PHE uptake.

**Conclusion**
Periodic health examination uptake is notably low in Jordan. Key determinants include recent visits to a primary healthcare facility, monthly income, and knowledge levels regarding PHEs and preventive health services. To enhance PHE participation, there is a critical need to integrate PHEs with primary healthcare services, increase awareness about PHEs, and offer free preventive services, particularly for high-risk groups.

**Keywords:**
Periodic health examination, PHE, preventive health services, routine health check-ups, Jordan, cross-sectional study.

**Introduction**
Routine Periodic Health Examinations (PHEs) for asymptomatic adults are integral to primary healthcare practice. These examinations involve clinical preventive services administered by primary healthcare clinicians to individuals without signs or symptoms of illness, constituting a routine healthcare process. The goal of these examinations is to proactively prevent morbidity and mortality by identifying modifiable risk factors and detecting early signs of treatable diseases.[1]

The Health Belief Model (HBM) was conceptualized to elucidate why individuals are reluctant to engage in disease prevention programs and health check-ups. As a crucial predictive framework, the HBM aids in understanding various health-related behaviors, including smoking, exercise, patient roles, and utilization of medical services.[2] Integrating with the HBM, health beliefs are defined as personal convictions associated with perceiving and managing specific diseases. These beliefs encompass key elements: perceived sensitivity, perceived severity, perceived benefit, perceived barrier, and cue to action.[3]

A systematic review recently published in the *Canadian Family Physician Journal* aimed to assess the reasons for visits to primary healthcare clinics. Clinicians participating in the review identified routine health maintenance as the third most prevalent reason for individuals seeking consultations with primary healthcare physicians. This ranking positioned routine health maintenance after upper respiratory tract infections and hypertension, highlighting the significant role of primary healthcare practitioners in motivating individuals to engage with preventive health services.[4]

A study conducted among undergraduate students in a Nigerian health science college found that 91.2% of participants demonstrated awareness of periodic health examinations (PHEs). However, actual participation in PHEs was notably low at 28.4%. The primary obstacles to uptake were identified as insufficient time, religious considerations, duration of education, perceived susceptibility to diseases, financial constraints, apprehension about the results, and a general lack of interest.[5]

A nationwide study in Saudi Arabia revealed that 22.9% of participants aged 15 years or older had undergone a Periodic Health Examination (PHE) in the preceding two years. The probability of receiving a PHE during this period exhibited positive correlations with various factors, including age, educational attainment, marital status, regular consumption of five servings of fruits and vegetables daily, diagnoses such as prediabetes, diabetes, or hypercholesterolemia, and visits to a healthcare setting within the last two years due to illness or injury.[6]

Jordan, classified as an upper-middle-income country, spans an area of 89,318 square kilometers and is divided into four provinces and 12 governorates. The population has grown substantially, increasing from 5.4 million in 2003 to over 11.5 million in 2023. This demographic shift can be attributed mainly to the influx of refugees and a relatively high birth rate.[7][8]

The country is undergoing a notable epidemiological transition characterized by a rising prevalence of non-communicable diseases (NCDs). These diseases are responsible for approximately 78% of deaths, establishing themselves as the primary cause of mortality and morbidity among the Jordanian population. Key risk factors contributing to the burden of NCDs include tobacco use, with a prevalence of about 50% (including e-cigarettes and shisha). One-quarter of the population reports insufficient physical activity and approximately 60% are classified as overweight or obese. Additionally, 22% of the population is hypertensive, 14% is diabetic, and the prevalence of depression is about 18%.[9]

This profile underscores a pressing concern regarding the country's high risk of non-communicable diseases (NCDs). There is a need for evidence-based preventive health measures to curb the progression of NCDs and their associated risk factors. If conducted according to evidence-based guidelines, periodic health examinations can effectively control both communicable and non-communicable diseases. Recognizing the urgency of the situation, gathering data on the uptake rate of periodic health examinations, and identifying the factors influencing this uptake is imperative. The absence of previous studies on the uptake of periodic health examinations in Jordan underscores the necessity for comprehensive research.

Our study aims to estimate the uptake of periodic health examinations among Jordanians while concurrently investigating various sociodemographic, health status, knowledge, and behavioral factors that influence this uptake. The findings from this research will not only contribute valuable insights into the current scenario but will also guide educational and promotional activities to encourage citizens to utilize preventive health services. In doing so, we strive to fill a crucial gap in existing knowledge and provide a foundation for evidence-based strategies to enhance public health in the country.

**Methodology**

**Recruitment**
This descriptive cross-sectional study was conducted using an anonymous web-based Google Forms questionnaire between 15 March and 1 May 2023. Due to limited resources, a convenience sampling method was employed to recruit participants. Jordanian residents aged 18 years or older who agreed to participate in our study were considered eligible. The research utilized a questionnaire with five key domains: sociodemographic information, health status, PHE uptake history, knowledge about periodic health examinations, and health behaviors based on the Health Belief Model (HBM). The questionnaire was distributed via WhatsApp and Facebook platforms, and participants were encouraged to share it with their family members. Additionally, data collection through face-to-face interviews targeted grand malls, mosques, and pharmacy clients, supplementing the online method.

The study adopted a stratified proportional sampling strategy across four provinces of Jordan. This approach was carefully designed to maintain a balance in gender and nationality among participants. The initial page of the online questionnaire explicitly outlined the study's objectives and provided detailed instructions on how to complete the questionnaire. The researcher's availability for clarifications ensured that participants' queries or doubts could be promptly addressed.

**Sampling Method**
**Inclusion Criteria:**

- Any citizen, regardless of nationality, aged 18 years or above and residing in Jordan.

**Exclusion Criteria:**

- Persons under 18 and individuals who declined to participate in the study.

A total of 362 respondents were recruited, aiming to provide a representative sample that reflects the entire population of Jordan in terms of district, age, sex, and nationality. The convenient sample size of 362 was calculated using the sample size formula for proportions:

N=Zα/22×P(1−P)D2N = Z_{\alpha/2}^2 \times \frac{P(1-P)}{D^2}N=Zα/22​×D2P(1−P)​

This calculation considered a study conducted in Saudi Arabia, where approximately 34% of the population underwent periodic health examinations.[10] The chosen values for statistical significance (α error) and margin of error (D) were 0.05 and 5%, respectively. As a result, the calculated sample size required for the survey was 345 respondents.

**Questionnaire Development**
The PHE questionnaire (Appendix 1), comprising 36 questions across five domains, was developed following an extensive literature review.[10][11][12][13][14] The questionnaire's five domains are as follows:

1. **Sociodemographic (9 items):** Inquires about relevant sociodemographic variables of participants.
2. **Health Status and Risk Factors (7 items):** Explores participants' health status and associated risk factors.
3. **PHE Uptake (4 questions):** Focuses on the outcome variable of periodic health examination.
4. **Knowledge about PHE and Preventive Health Services (8 items):** Assesses knowledge using a 3-option scale ('agree,' 'don’t agree,' 'I don’t know'). The items are scored, with correct answers receiving a score of 1 and incorrect or 'I don’t know' responses scoring 0. The total score ranges from 0 to 8, with higher scores indicating greater knowledge of health check-ups and preventive measures. Cronbach’s α, estimated during the pilot phase with 25 participants, was 0.68.
5. **Health Behaviors towards PHE based on HBM (6 items):** Measures health behaviors using a 5-point Likert scale ranging from 1 ('strongly disagree') to 5 ('strongly agree'). The total scores range from 6 to 30, with higher scores indicating more positive health beliefs for each item. The Cronbach’s α for health behaviors towards PHE during the pilot testing phase was 0.74, demonstrating acceptable internal consistency.

The questionnaire was translated into Arabic for comprehensibility and then back-translated into English with the assistance of an expert translator. This rigorous process ensured the questionnaire's clarity and accuracy across languages.

**Statistical Analysis**
The primary outcome variable is the uptake of periodic health examinations in Jordan, categorized as a dichotomous (yes or no) variable. The independent variables encompass sociodemographic, health status, knowledge, and health behavioral factors. Records with missing data were excluded to ensure the integrity of the analysis. Data were analyzed using IBM SPSS, version 26.0 (IBM, Armonk, NY, USA).

Participant characteristics were examined using counts, percentages, means, and standard deviations (SD) through descriptive statistics. Graphs and tables were employed as needed for visual representation. A 95% confidence interval was calculated using appropriate methods, and a 2-sided p-value less than .05 was considered statistically significant.

A binary logistic regression test was used to study the association between the binary outcome variable and the various continuous and nominal predictor variables. Multivariate logistic regression analysis was employed to examine the relationship between the uptake of periodic health examinations and various independent co-variables to adjust for confounding.

A hierarchical block-wise logistic regression model was also constructed to identify the most potent predictor variables. This comprehensive approach blends descriptive, inferential, and multivariate statistical techniques to provide a thorough understanding of the factors influencing the uptake of periodic health examinations in Jordan.

**Ethical Considerations**
Before the formal survey, the study protocol was approved by the Jordan University Ethics Committee (approval number: 13-2023) and the Jordan University Hospital (approval number: 10/2023/4560). The questionnaire was designed to be anonymous and voluntary, and respondents were informed that submission of the questionnaire implied informed consent. The data were kept confidential, and the results did not identify the respondents personally. Contact information for the researcher was provided for clarification purposes.

**Results**

Three hundred sixty-five individuals participated in the study between March and April 2023. Three participants were excluded (one was under 18 years old, and the other two did not complete the questionnaire), leaving 362 participants for analysis.

**Descriptive Statistics**
The demographic characteristics of participants are summarized in Table 1. The mean age was 38.2 years (range 18 to 88 years, SD 14.6). Of the 362 participants, slightly more than half were male (185, 51.1%). Approximately 230 participants (63%) were married, 270 (74.6%) were Jordanians, and 202 (55.8%) held a university degree. Most participants (225, 62.2%) reported a monthly income of less than 500 Jordanian dinars (700 USD), and half lacked health insurance.

**Table 1: Sociodemographic Characteristics of Participants (N=362) in the Study "Periodic Health Examination in Jordan," 2023**

| **Characteristic** | **Participants; n (%)** |
| --- | --- |
| **Gender** |  |
| Male | 185 (51.1) |
| **Age group (years)** |  |
| 18-29 | 122 (23.7) |
| 30-39 | 90 (24.9) |
| 40-49 | 70 (19.3) |
| 50-59 | 41 (11.3) |
| ≥60 | 39 (10.8) |
| **Marital status** |  |
| Married | 230 (63.5) |
| Single | 101 (27.9) |
| Divorced | 14 (3.9) |
| Widowed | 17 (4.7) |
| **Monthly income (JDs)**a |  |
| <500 | 225 (62.1) |
| 500-999 | 93 (25.7) |
| 1000-1499 | 26 (7.2) |
| 1500-1999 | 10 (2.8) |
| ≥2000 | 8 (2.2) |
| **Educational level** |  |
| Elementary school | 42 (11.6) |
| Secondary school | 118 (32.6) |
| University | 166 (45.9) |
| Postgraduate | 36 (9.9) |
| **Province of residence** |  |
| Amman | 151 (41.7) |
| Central Jordan | 82 (22.7) |
| North Jordan | 100 (27.9) |
| South Jordan | 29 (8.0) |
| **Nationality** |  |
| Jordanians | 270 (74.6) |
| Syrians | 47 (13.0) |
| Palestinians | 22 (6.0) |
| Egyptians | 18 (5.0) |
| Iraqis | 5 (1.4) |

*Note:* aJD = Jordanian dinars

Regarding health status, Table 2 shows that 240 participants (66.3%) reported good or excellent health, 78 (21.5%) had a chronic disease, and 200 (55%) visited a primary healthcare clinic in the past year. Additionally, 191 participants (52.8%) were current smokers.

**Table 2: Health Characteristics of Participants in the Study "Routine Health Checkups in Jordan," 2023**

| **Variable** | **Participants; n (%)** |
| --- | --- |
| **VPHCFa within the last year** |  |
| Yes | 200 (55.2) |
| No | 162 (44.8) |
| **NCDsb** |  |
| Yes | 78 (21.5) |
| No | 240 (78.5) |
| **Smoking** |  |
| Smoker | 191 (52.8) |
| Non-smoker | 171 (47.2) |
| **Health insurance** |  |
| Insured | 183 (50.6) |
| Not insured | 179 (49.4) |
| **Seasonal flu vaccination** |  |
| Yes | 60 (18.6) |
| No | 302 (83.4) |
| **Health status self-evaluation** |  |
| Poor | 9 (2.5) |
| Fair | 25 (5.9) |
| Good | 88 (24.8) |
| Very good | 136 (37.6) |
| Excellent | 104 (28.7) |
| **BMIc ≥ 25** |  |
| Yes | 223 (61.6) |
| No | 139 (38.4) |
| **Physical activity** |  |
| Yes | 108 (29.8) |
| No | 254 (70.2) |

*Notes:*
aVPHCF = Visit to Primary Healthcare Facility
bNCD = Noncommunicable Diseases
cBMI = Body Mass Index

Regarding periodic health examination (PHE), only 98 of the 362 participants (27.1%, 95% CI 22.8 - 31.9) underwent a medical check-up in the last two years.

**Logistic Regression Analysis**
When analyzing the predictor factors associated with the uptake of Periodic Health Examination (PHE), the Forest plot in Figure 1 highlights several significant findings.

**Figure 1: Univariate Logistic Regression Analysis for Predictor Factors of PHE Uptake, Jordan 2023**

1. **Age**: Age played a significant role, revealing that with each additional year, the odds of undertaking PHE increased by 2.2% (P = .006, OR = 1.022, 95% CI 1.006 - 1.038).
2. **Nationality**: Nationality also proved to be a factor, with Syrians demonstrating a lower frequency of PHE uptake. The odds of Syrians undergoing PHE were 0.283 compared to Jordanians (P = .01, OR = 0.28, 95% CI 0.11 - 0.74).
3. **Education Level**: Education level exhibited a strong association, with postgraduates displaying more than six times the odds of undertaking PHE than individuals with only primary school education (P = .001, OR = 6.62, 95% CI 2.12 - 20.71).
4. **Occupation**: Healthcare workers displayed more than twelve times the odds of undergoing PHE than general employees (P < .001, OR = 12.28, 95% CI 4.69 - 32.19).
5. **Monthly Income**: Individuals earning more than 2000 JD monthly have twelve times the odds of receiving PHE compared to those with a monthly income of less than 500 JD (P = .003, OR = 12.00, 95% CI 2.34 - 61.45).
6. **Health Insurance**: Health insurance emerged as a significant facilitator of PHE uptake. Insured participants demonstrated more than two times the odds for undertaking PHE than non-insured individuals (P = .001, OR = 2.30, 95% CI 1.42 - 3.71).
7. **Chronic Diseases**: People with chronic diseases have more than twice the odds of undertaking PHE than those without chronic disease (P = .005, OR = 2.3, 95% CI 1.258 - 3.629).
8. **Visits to Primary Healthcare Clinics**: Visits to a primary healthcare clinic in the past year significantly impacted PHE uptake. They have five times the odds of PHE uptake compared to those who did not visit a primary healthcare facility in the past year (P < .001, OR = 4.91, 95% CI 2.82 - 8.57).
9. **Physical Activity**: Participants who are physically active have 1.65 times the odds of undertaking PHE than those without enough physical activity (P = .046, OR = 1.65, 95% CI 1.01 - 2.69).
10. **Health Knowledge**: For every extra point in knowledge about periodic health examinations, there is a 39% increase in PHE uptake (P < .001, OR = 1.39, 95% CI 1.18 - 1.64).

On the other hand, several variables were not associated with periodic health examination uptake. These included gender (P = .334), smoking status (P = .759), marital status (P = .524), health status self-evaluation (P = .184), seasonal influenza vaccination (P = .069), health behavior factors combined (P = .340), and BMI (P = .758).

**Adjusted Logistic Regression Model**
After adjusting for confounding variables and selecting clinically and statistically significant factors, a logistic regression model was constructed

**Discussion**

**Principal Findings and Comparison with Other Studies**

**Only 98 out of 362 participants (27.1%) underwent a Periodic Health Examination (PHE) in the last two years, with a 95% confidence interval ranging from 22.8% to 31.9%. Significant predictors of PHE uptake include recent visits to a primary healthcare facility, monthly income, and knowledge about PHE and preventive health measures. Non-significant factors, including gender, marital status, smoking status, and BMI, did not show a significant association with PHE uptake.**

**Interestingly, our observed uptake rate is comparable to findings from studies conducted in Saudi Arabia [6], [10] and Nigeria [12]. However, it notably falls below the rates reported in studies conducted in the United States [1], the United Kingdom [13], and Switzerland [15]. These comparative statistics highlight variations in PHE utilization across different regions, suggesting potential differences in healthcare practices, accessibility, or public awareness. The similarities in PHE uptake between Jordan, Saudi Arabia, and Nigeria could indicate shared regional trends, while the disparities with the USA, UK, and Switzerland may stem from distinct healthcare systems, cultural influences, or healthcare policies.**

**Our study identified the most influential determinant of PHE uptake as a recent visit to a primary healthcare facility within the last year. This finding aligns with consistent results observed in several studies [6], [16], [17]. Notably, individuals who had visited primary healthcare clinics in the last year were found to be five times more likely to undergo PHE compared to those who did not have such recent visits. This significant association persisted even after adjusting for other relevant factors, underlining the robustness of this relationship.**

**The second critical factor influencing PHE uptake was monthly income, a result that is consistent with findings from various studies [1], [12], [14], [17], [18], [19], [20], [21]. The impact of monthly income on PHE uptake underscores the role of socio-economic factors in shaping healthcare-seeking behaviors, emphasizing the need for targeted interventions to address disparities.**

**The third influential factor was knowledge about PHE, consistent with previous research [22], [23], [24]. This finding highlights the importance of informed decision-making in healthcare utilization. It is worth noting that knowledge about PHE is intertwined with other factors such as educational level and occupation. However, even after adjusting for these related factors, the association between knowledge about PHE and uptake remained robust.**

**Several additional variables were associated with PHE uptake, providing a nuanced understanding of factors influencing preventive healthcare practices:**

- **Age: We found a positive association between age and PHE uptake, aligning with results from other studies [13], [17], [19]. This suggests that as individuals get older, they are more likely to engage in routine health examinations, possibly due to the increased prevalence of non-communicable diseases (NCDs) in older age or a greater emphasis on preventive care with advancing age.**
- **Nationality: Notably, individuals of Syrian nationality were found to be less likely to undergo PHE than Jordanians. Economic factors may contribute to this difference, emphasizing the need for targeted interventions to ensure equitable access to preventive healthcare services among diverse populations.**
- **Place of Residence: Individuals residing in the North region were less likely to undergo PHE than those in Amman city. This regional disparity suggests potential variations in healthcare accessibility or awareness, highlighting the importance of tailoring interventions to specific locations.**
- **Educational Level: Educational attainment exhibited a strong association with PHE uptake, with a noticeable increase in uptake corresponding to higher levels of education. This finding is consistent with results from other studies [17], [21], [25].**
- **Occupation: Healthcare workers and retired individuals were more likely to undergo PHE than general employees. The higher likelihood of PHE uptake among healthcare workers may be attributed to a heightened awareness of the importance of preventive healthcare within this group. Age may be a confounding factor for retired individuals, influencing both retirement status and PHE uptake.**

**Our study identified several health-related factors associated with PHE uptake. These factors include the presence of chronic diseases supported by other studies [6], [14], [18], [22], [26], being insured supported by studies [17], [21], [25], [27], [28], and engagement in physical activity [1], a correlation consistent with numerous studies. Understanding these health-related determinants provides valuable insights for healthcare providers and policymakers seeking to enhance preventive healthcare practices.**

**Factors Not Significantly Associated with PHE Uptake:**

**These include gender, contrary to numerous studies that found females more likely to undertake PHE than males [6], [13], [15], [20]. Marital status, being married, is often linked to higher PHE uptake in previous studies [1], [13], [14], [15], [19], [29], [30]. Surprisingly, smoking status was not found to be associated with PHE uptake, contrary to findings from several studies that suggested smokers are less likely to undergo PHE than non-smokers [11], [13], [15], [20], [29]. These non-associated factors highlight the complexity of health behavior determinants and suggest variations within the specific context of the studied population.**

**Our study found no significant association between combined behavioral factors and PHE uptake, contradicting findings in many studies [3], [11], [14], [20], [30], [31]. Possible explanations include the questionnaire's suitability for the Jordanian population or participant comprehension issues, emphasizing the need for culturally sensitive research methodologies and precise survey instruments in future studies.**

**Strengths of the Study**

1. **First of Its Kind in Jordan: This study fills a gap in existing knowledge by being the first to investigate PHE uptake in Jordan.**
2. **Comprehensive Analysis: The study employs a robust methodology, combining descriptive, inferential, and multivariate statistical techniques to provide a thorough understanding of PHE uptake.**
3. **Significant Predictors: Key factors influencing PHE uptake were identified, offering valuable insights for healthcare providers and policymakers.**

**Limitations of the Study**

1. **Cross-Sectional Design:**
   - **Limitation: The study's design limits the ability to establish causality.**
   - **Improvement: Future research could benefit from a longitudinal approach to better establish causal relationships between the identified predictors and PHE uptake.**
2. **Convenient Sampling:**
   - **Limitation: This method may introduce selection bias, and the online survey format may lead to measurement bias.**
   - **Improvement:**
     - **A stratified sampling method was employed, taking a sample from each of the four provinces of Jordan proportional to population size and stratifying the sample according to gender, age group, and nationality (Table 1).**
     - **A hybrid online and face-to-face interviewing approach was integrated.**
     - **The sample was collected from diverse settings: social media platforms, grand malls, mosques, and pharmacy clients.**
     - **The author was available for clarification through WhatsApp and email.**
3. **Survey Instrument:**
   - **Limitation: The questionnaire’s comprehensiveness and relevance to the Jordanian context might not have been fully ensured.**
   - **Improvement:**
     - **A pilot study with 25 participants was conducted, and the questionnaire was adjusted according to feedback and reliability measures. In future research, a larger sample for the pilot study is advised.**
4. **Behavioral Factors:**
   - **Limitation: The study did not find a relationship between behavioral factors and PHE uptake, which contradicts findings in other contexts.**
   - **Improvement: To clarify these results in future research, a more detailed investigation into cultural and societal influences on health behaviors in Jordan is needed.**
5. **Limited Generalizability:**
   - **Limitation: Results may not be generalizable to populations outside of Jordan or those not included in the sample.**
   - **Improvement: Expanding future studies to include diverse populations and different geographic regions would provide a more comprehensive understanding of PHE uptake.**

**Future Directions**

1. **Integration of Preventive and Primary Health Care Services: The most significant predictor of PHE uptake is a recent visit to the Primary Health Care Facility (PHCF). We recommend integrating preventive health services with primary healthcare to enhance accessibility and efficiency [32], providing incentives for both healthcare providers and patients [33].**
2. **Offering Free Preventive Services: To address the impact of economic factors on PHE uptake, we propose integrating free preventive services within the primary healthcare context. Negotiations with private health insurance companies to include preventive services, including PHE, in their coverage can further promote accessibility [33].**
3. **Increasing Awareness through Campaigns: The positive association between knowledge about PHE and uptake suggests a need for increased awareness. Organized and evidence-based campaigns can effectively raise awareness about PHE and preventive measures in general.**
4. **Exploring Behavioral Factors in Jordan: Our study found no relationship between behavioral factors (sensitivity, self-efficacy, perceived severity, and perceived benefit) and PHE uptake. Given that other studies have identified such relationships, we recommend further investigating these factors in the Jordanian context in future studies.**
5. **In-depth Evaluation of Smoking as a Predictor: Our study suggests that smoking was not associated with PHE uptake, contrary to findings in other studies. To better understand the nuances and contributing factors, we recommend conducting more detailed and specific studies on smoking as a predictor factor for PHE uptake in the Jordanian population.**

**Conclusion:**
Periodic health examination uptake is notably low in Jordan. Key determinants of this uptake include recent visits to a primary health care facility within the last year, monthly income, and knowledge levels regarding periodic health examination (PHE) and preventive health services. To enhance PHE participation, there is a critical need for the integration of periodic health examinations with primary health care services, increased awareness about PHE, and offering free preventive services, particularly for those at high risk.
